# Self-organization of mortal filaments and its role in bacterial division ring formation

**DOI:** 10.1038/s41567-024-02597-8

**Published:** 2024-08-12

**Authors:** Christian Vanhille-Campos, Kevin D. Whitley, Philipp Radler, Martin Loose, Séamus Holden, Anđela Šarić

**Affiliations:** 1 Institute of Science and Technology Austria, Klosterneuburg, Austria; 2 Department of Physics and Astronomy, Institute for the Physics of Living Systems, University College London, London, UK; 3 Centre for Bacterial Cell Biology, Biosciences Institute, Newcastle University, Newcastle upon Tyne, UK; 4 Archaea Biology and Ecogenomics Unit, Department of Functional and Evolutionary Ecology, University of Vienna, Vienna, Austria; 5 School of Life Sciences, The University of Warwick, Coventry, UK

**Keywords:** Biological physics, Computational biophysics

## Abstract

Filaments in the cell commonly treadmill. Driven by energy consumption, they grow on one end while shrinking on the other, causing filaments to appear motile even though individual proteins remain static. This process is characteristic of cytoskeletal filaments and leads to collective filament self-organization. Here we show that treadmilling drives filament nematic ordering by dissolving misaligned filaments. Taking the bacterial FtsZ protein involved in cell division as an example, we show that this mechanism aligns FtsZ filaments in vitro and drives the organization of the division ring in living *Bacillus subtilis* cells. We find that ordering via local dissolution also allows the system to quickly respond to chemical and geometrical biases in the cell, enabling us to quantitatively explain the ring formation dynamics in vivo. Beyond FtsZ and other cytoskeletal filaments, our study identifies a mechanism for self-organization via constant birth and death of energy-consuming filaments.

## Abstract (web summary)

Treadmilling of cytoskeletal filaments is crucial for their functional self-organization. Now the mechanism underpinning this collective organization is shown to be the dissolution of misaligned filaments.

## Main

Cytoskeletal filaments are active cellular elements that continuously grow and shrink through addition and removal of subunits. One prominent example of this activity is treadmilling—a process by which protein filaments grow on one end while the other end shrinks at an equal rate. This dynamic behaviour is driven by nucleotide hydrolysis and results in the filaments seemingly moving, despite the individual filament monomers remaining in one place^1^. Many essential cytoskeletal filaments, such as actin, microtubules and FtsZ, exhibit this important feature^2–6^.

From a physics point of view, treadmilling has been often modelled as self-propulsion, where the filament centre of mass moves directionally with a certain velocity to mimic the directional filament growth^7–9^. While this approach can be appropriate for describing single-filament dynamics, when it comes to the assembly into higher order dynamic structures, self-propelled models might not be suitable. Such models fail to capture the characteristic constant turnover of components in treadmilling systems and by introducing propelling forces may overestimate the forces generated by filament polymerization on obstacles, which have been shown to be small or negligible^10^ and instead likely operate via a ratchet-like interplay between polymerization and thermal fluctuations^11^. The self-organization mechanisms of treadmilling polymers may hence be different from those of self-propelled filaments and remain unexplored.

Here we develop a computational model for the collective behaviour of treadmilling filaments, accounting for the kinetics of nucleation, growth and shrinkage, to study their collective dynamics. We focus on investigating the self-organization of FtsZ filaments, a highly conserved tubulin homologue^12^, widely present in bacteria and one of the best characterized treadmilling systems in the literature^13–16^. Our model allows us to explore the interplay between polymerization dynamics and collective filament organization at the cellular scale and to directly compare to in vitro and in vivo experiments. Treadmilling FtsZ filaments self-organize into a dynamic ring^17^ in the middle of bacterial cells (the ‘Z-ring’) that orchestrates bacterial cell division^18–20^. Treadmilling has been shown to be essential for cell division of *Escherichia* *coli*, *Bacillus* *subtilis*, *Staphylococcus aureus* and *Streptococcus pneumoniae*^15,16,21,22^, and in *B. subtilis*, it has been shown to be required for the formation of a coherent Z-ring^23^. However, how treadmilling contributes to the self-organization of individual filaments into a large-scale cytoskeletal structure remains unclear.

We first show that our model correctly reproduces single-filament treadmilling dynamics collected in vitro^13^ and proposed in previous theoretical kinetic studies^24^. We then demonstrate that treadmilling polymers collectively align due to shrinkage and ‘death’ of misaligned filaments, yielding quantitative matching with high-speed atomic force microscopy (HS-AFM) imaging of reconstituted FtsZ filaments. Finally, we find that such a system forms tight ordered dynamic rings when under external biases present in bacterial division. The model quantitatively explains the time-dependent in vivo dynamics of Z-ring condensation and maturation^23,25^ in *B. subtilis*. These results identify a mechanism for ordering of cytoskeletal filaments through dynamic growing and shrinking, which is responsible for the formation of the bacterial division ring and potentially present in a plethora of other cytoskeletal processes across the tree of life.

### Model description

**Fig. 1:**
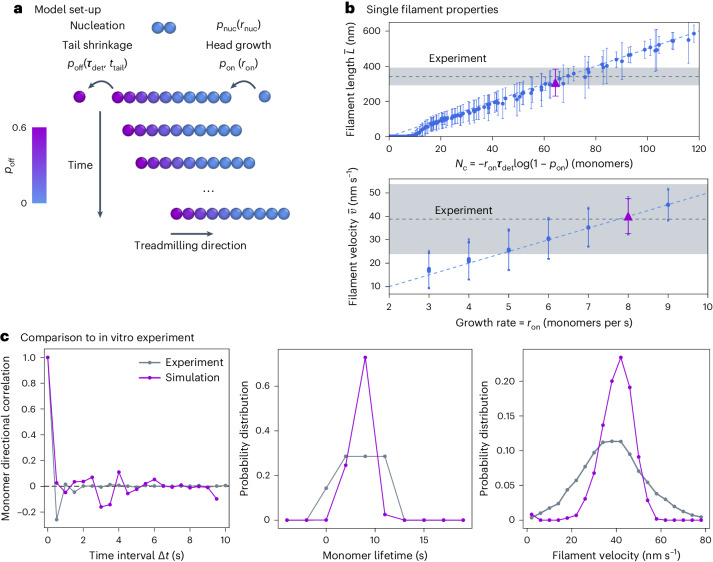
Coarse-grained model for treadmilling filaments. **a**, New filaments nucleate at a constant rate *r*_nuc_, grow with probability *p*_on_ = *r*_on_d*t* and shrink with probability *p*_off_, determined by the monomer detachment time ${\tau }_{\det }$ and the time the monomer has been in the system. This growth and shrinkage dynamics results in the apparent directional motion of the filaments over time. **b**, Upper, single filament length against the corresponding intrinsic size *N*_c_. The blue dashed line is the theoretical $\bar{L}={N}_{{{{\rm{c}}}}}\sigma$ (*σ* = 5 nm). Lower, average treadmilling velocity $\bar{v}$ of a filament against the corresponding growth rate *r*_on_. The blue dashed line is the theoretical $\bar{v}={r}_{{{{\rm{on}}}}}\sigma$. In both graphs, each point is the steady-state average over 20 replicas of single-filament simulations with error bars for the standard error of the average. The grey dashed lines correspond to the fluorescence microscopy experiments of in vitro reconstituted *E. coli* FtsZ treadmilling filaments on SLBs at 1.25 μM (shaded areas for error). Purple triangles correspond to *r*_on_ = 8 monomers per second and ${\tau }_{\det }=5$ s, which fit experimental measurements best and are used for the simulation data in **c**. **c**, Single-filament dynamics in simulations (in purple) quantitatively matches in vitro FtsZ dynamics (in grey). Left, directional autocorrelation of FtsZ monomers at increasing time intervals Δ*t* decays quickly, showing the static nature of individual monomers. Middle, monomer lifetime distribution. Right, treadmilling filament velocity distribution.

We model treadmilling filaments as coarse-grained polymers made of spherical beads with diameter *σ* (the simulation unit of length) on a two-dimensional plane with periodic boundary conditions^26^. Individual beads correspond to distinct monomers, with *σ* = 5 nm as known for FtsZ^19,27,28^. Filaments are formed by connecting monomers via harmonic springs and angle potentials to capture filament stiffness (see Supplementary Information for more details). We time-evolve our system in molecular dynamics simulations and impose filament nucleation, growth and shrinkage kinetics by dynamically creating and deleting monomers in the system (Fig. 1a and Supplementary Video 1; see Supplementary Information for more details).

Treadmilling filaments are active, dissipating energy via nucleotide hydrolysis at the monomer–monomer interface. This results in a change in the interface domains of the monomers, which then tend to dissociate when they reach the tail of the filament^24,29–31^. To capture these properties, we consider reactions at time intervals d*t*_react_ = 0.1 s during which individual filaments grow and shrink with probabilities *p*_on_ and *p*_off_ respectively, and new filaments are nucleated with probability *p*_nuc_. Treadmilling depends only on two parameters: the head polymerization rate, *r*_on_, and the typical time over which monomers become available for detachment as a result of hydrolysis, ${\tau }_{\det }$. Here *r*_on_ (in monomers per second) captures both the bulk free monomer concentration, which we assume is constant, and the polymerization rate constant, such that *p*_on_ = *r*_on_d*t*_react_. Monomers in solution are thus implicitly considered. Importantly, filament polymerization is allowed only if free space for monomer addition is available around the filament head. Tail depolymerization is modelled through ${p}_{{{{\rm{off}}}}}=1-{\rm{e}}^{-{t}_{{{{\rm{tail}}}}}/{\tau }_{\det }}$, where ${\tau }_{\det }$ (in seconds) effectively accounts for both the slow nucleotide hydrolysis at the monomer–monomer interface in the filament and the fast monomer dissociation at the filament tail, while *t*_tail_ is the time for which the tail monomer has been in the system (see Supplementary Fig. 3 for a detailed exploration of the hydrolysis and dissociation reactions). Given that monomers detach into solution upon depolymerization, *p*_off_ is not dependent on the environment of the filament on the surface^32^. To reach steady state, *p*_on_ = *p*_off_ must be satisfied, which defines an intrinsic size ${N}_{{{{\rm{c}}}}}=-{r}_{{{{\rm{on}}}}}{\tau }_{\det }\,\log (1-{p}_{{{{\rm{on}}}}})$ around which filaments fluctuate while treadmilling at constant velocity *v*_c_ = *r*_on_*σ*. This also defines a typical monomer lifetime ${\tau}_{{{{\rm{c}}}}}=-{\tau }_{\det }\,\log (1-{p}_{{{{\rm{on}}}}})$. Finally, insertion of new filament nuclei into the system (modelled as dimers) is controlled by the imposed nucleation rate *r*_nuc_ (in nuclei per second), such that *p*_nuc_ = *r*_nuc_d*t*_react_. Like the growth rate, *r*_nuc_ captures both the nucleation rate constant and the free monomer concentration.

### Single-filament dynamics

We first perform simulations of individual filaments for a range of filament growth and detachment time parameter sets $\{{r}_{{{{\rm{on}}}}},{\tau }_{\det }\}$. We initialize the system with a single filament nucleus and let it evolve in time (Supplementary Video 1). As expected, filaments reach a steady state where they fluctuate around a constant length and treadmilling velocity (Fig. 1b), while the individual monomers display finite lifetimes (Supplementary Figs. 4 and 5). Experimentally, FtsZ protofilaments have been measured (in vivo and in vitro) to be between 100 and 500 nm long and to treadmill at speeds between 15 and 50 nm s^−1^, while individual monomers turnover at lifetimes around 8 s, depending on the bacterial strain and conditions^25,28,33–35^, all of which are well within the range we observe in simulations (Fig. 1b).

We now turn to a direct comparison of our model with experiments. For this purpose, we reconstituted *E. coli* FtsZ filaments (FtsA and fluorescently labelled FtsZ) on supported lipid bilayers (SLBs) with excess adenosine triphosphate and guanosine triphosphate and used total internal reflection fluorescence microscopy to image treadmilling dynamics and filament organization (see Supplementary Information for further details). Using previously developed image analysis software^36,37^ we measure filament lengths and velocities (grey dashed lines and shaded regions in Fig. 1b, also reported in ref. ^36^) as well as individual monomer lifetimes (Supplementary Figs. 4 and 5). When choosing $\{{r}_{{{{\rm{on}}}}},{\tau }_{\det }\}$ model parameters that match this specific experimental system (the purple triangles in Fig. 1b), the velocity and lifetime distributions also display a good agreement with the experimental data (Fig. 1c, middle and right).

Importantly, the directional autocorrelation function of individual monomers for different time intervals Δ*t*, measured both in simulations and in in vitro experiments, presents a sharp decay to zero (Fig. 1c, left), indicating that individual monomers remain static even though the filament as a whole appears to be moving. The same is also clearly visible from the individual molecule track analysis in vitro (Supplementary Fig. 7). This lack of motility is an important feature of treadmilling filaments, which also suggests that modelling treadmilling as self-propulsion is likely unsuitable, as individual monomers do not display any directional motion. Consequently, pushing forces exerted by treadmilling filaments might be small, as previously discussed for actin bundles^10^. Together, these results demonstrate that our model correctly captures the main aspects of treadmilling dynamics on biologically relevant time and length scales and that it can be benchmarked for a specific protein under particular conditions and used for quantitative comparison with experiments.

### Collective filament dynamics

**Fig. 2:**
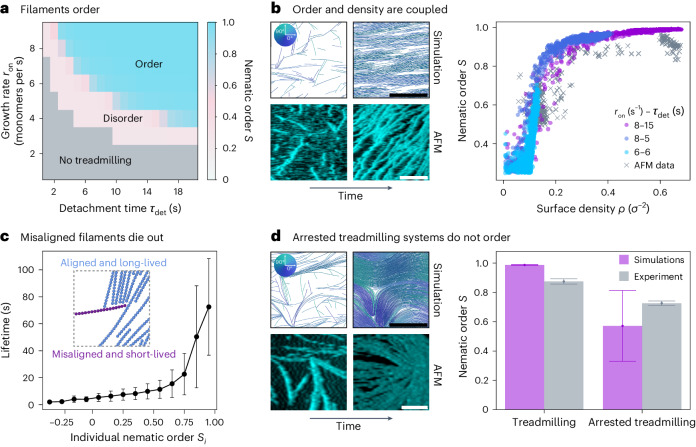
Collective behaviour of treadmilling filaments. **a**, Steady-state nematic order parameter *S* measured in simulations (average over *N* = 10 replicas, *t* > 10 min). Grey indicates the quasi-empty systems of non-treadmilling filaments, blue the nematically ordered system with polar bands and pink the disordered transitional region. **b**, Left, snapshots of treadmilling systems at early (leftmost column) and late (rightmost column) times in simulations and in HS-AFM experiments (filaments are coloured according to their orientation—see the colour wheel in the inset). Right, nematic order *S* plotted against surface density *ρ* at different points in time for reconstituted FtsZ on SLBs (AFM data is shown as crosses) and simulations (coloured according to kinetics and shown as circles). For experiments, data from *N* = 3 different videos is shown. For simulations, *N* = 10 different trajectories are shown for each parameter set. **c**, Filament lifetime for different levels of alignment (characterized by the individual nematic order *S*_*i*_). We consider only simulations for *N*_c_ ≥ 100 (*N* = 10 replicas per parameter set), which results in 226,077 data points (filaments) in total, binned in *S*_*i*_ with width 0.1. Points correspond to the mean of the bin and the error bar to the standard deviation. Inset, snapshot of a trapped misaligned filament (purple) that dissolves over time (Supplementary Video 7). **d**, Left, snapshots of systems with arrested treadmilling at early (leftmost column) and late (rightmost column) times in simulations (same kinetic parameters as in **b** and in HS-AFM experiments). Right, steady-state nematic order *S* for treadmilling and non-treadmilling systems. Bars and dots indicate the average for *t* > 600 s (*N* = 10 replicas in simulations: *n* = 600 data points in simulations, *n* = 28 data points for treadmilling experiments and *n* = 48 for arrested treadmilling experiments), and error bars indicate the standard deviation. In experiments, ‘treadmilling’ corresponds to wild-type data and ‘arrested treadmilling’ to the L169R mutant. Unless stated otherwise, simulation data corresponds to *r*_on_ = 8 s^−1^, ${\tau }_{\det }=15\,{{{\rm{s}}}}$ and *r*_nuc_ = 1 s^−1^ for *l*_p_ = 10 μm and *D* = 100 nm^2^ s^−1^. Scale bars, 500 nm.

We are now in a position to explore the collective dynamics of many treadmilling filaments and how their mortality affects the formation of higher order structures. We nucleate filaments in the system, characterized by parameters $\{{r}_{{{{\rm{on}}}}},{\tau }_{\det }\}$, at a constant nucleation rate *r*_nuc_. The system generally reaches steady-state surface density *ρ* and average filament size $\bar{N}$ while the filament population undergoes continuous turnover (Supplementary Fig. 8). We observe three different collective regimes (Fig. 2a), which depend exclusively on the treadmilling kinetics or the resulting intrinsic filament size *N*_c_ (Supplementary Figs. 8–10).

The first regime occurs for very low values of the intrinsic filament size *N*_c_ (grey area in Fig. 2a), where individual filaments die out rapidly (Supplementary Fig. 6), resulting in a quasi-zero surface density (Supplementary Figs. 8–10). As the intrinsic filament size increases (*N*_c_ ≈ 10) the system transitions into an unstable treadmilling regime, where filament size fluctuations are comparable to their size (Supplementary Fig. 6). The system is then populated by treadmilling filaments that stochastically emerge and disappear, never reaching high surface densities (pink region in Fig. 2a and Supplementary Video 2). Finally, for higher values of *N*_c_ (*N*_c_ ≫ 10), the intrinsic size fluctuations become negligible compared to the filament size (Supplementary Fig. 6), and filaments enter a highly stable and persistent treadmilling regime in which they can achieve long lifetimes. This allows the system to build up its filament population and reach higher surface densities (Supplementary Figs. 8–10).

We find that the majority of this parameter space is characterized by the emergence of a large-scale nematic order (Fig. 2a,b) and the formation of polar bands (snapshots in Fig. 2b and Supplementary Video 3). This behaviour is highly reminiscent of the nematic laning phase previously observed in self-propelled rods^38,39^ and is similarly characterized by high values of local and global nematic order parameter *S* and high local but low global values of the polar order parameter due to lanes formation (Supplementary Fig. 13). Unlike in self-propelled^8,38,39^ or passive^40,41^ nematic systems, where the system density determines the ordering, in a treadmilling system the turnover kinetics (characterized by *N*_c_) dictates the emergent steady-state ordering and density (Fig. 2b and Supplementary Fig. 10). We find that if monomer hydrolysis and hence treadmilling are arrested and the filaments grow long, the system will evolve towards a highly populated but disordered configuration (Fig. 2d, Supplementary Fig. 15 and Supplementary Video 4). This occurs because, while thermal fluctuations and collisions can foster local alignment and bundling, the system cannot resolve the nematic defects that stochastically arise as filaments nucleate. Hence, treadmilling serves as an effective mechanism for defect healing.

We now turn to a comparison of collective ordering with experiment. As shown in Fig. 2b, HS-AFM images of *E. coli* FtsZ filaments on a SLB bear a striking resemblance to simulation snapshots, with an evident transition to a high nematic order as the system becomes more populated (Supplementary Video 5). These high-resolution images allow us to quantify both the orientational order and the surface density of the system at any time from vector field analysis (Supplementary Fig. 16), enabling a quantitative comparison with the simulations. Figure 2b demonstrates that both HS-AFM and simulation trajectories display the same coupling between orientational order and density, independent of the specific kinetic parameters used. Furthermore, when we perform HS-AFM with a FtsZ mutant with reduced GTPase activity and greatly inhibited depolymerization (FtsZ L169R)^9^, we observe that, just like in simulations, nematic defects are not resolved over time and the system remains at lower nematic order than wild type (Fig. 2d and Supplementary Video 6). These results indicate that our model correctly describes FtsZ treadmilling behaviour as observed experimentally and should therefore provide insight into the mechanisms underlying filament ordering.

In simulations, we find that the ordering transition is driven by filaments growing against each other, which eventually selects for a common alignment direction, like in systems of self-propelled rods^38,39,42^. However, the underlying mechanism is completely different: unlike self-propelled rods that exert force when collided with another filament^43–46^, treadmilling filaments stop growing when they reach another object, but still continue to depolymerize, which results in shrinkage, reorientation or even dissolution of the filaments. Ultimately, the fact that shrinkage is independent of growth causes misaligned filaments to die out, because they are more likely to grow against the neighbours than their aligned counterparts. Supplementary Video 7 exemplifies a striking example of such a filament which, by being misaligned with its surroundings, stops growing and eventually dies.

Figure 2c measures the lifetime of each filament against its average alignment, characterized by the individual nematic order *S*_*i*_. These two variables are indeed highly correlated: only very aligned filaments (*S*_*i*_ ≈ 1) reach long lifetimes, up to several minutes, while strongly misaligned polymers die out after a few seconds on average and are gradually replaced with aligned ones. The aligned filaments also treadmill with a velocity that approaches their single-filament value *v*_c_ = *r*_on_*σ*, while the misaligned filaments typically have vanishing velocities (Supplementary Fig. 11). Globally, this behaviour results in the progressive selection of the more stable filaments that align with the rest of the system, reminiscent of what was previously proposed for plant microtubules^47,48^. The filament death and birth mechanism and the establishment of order are not rapid but require turnover of a large number of filaments and typically take minutes (Supplementary Fig. 12).

### Treadmilling drives bacterial division ring condensation

**Fig. 3:**
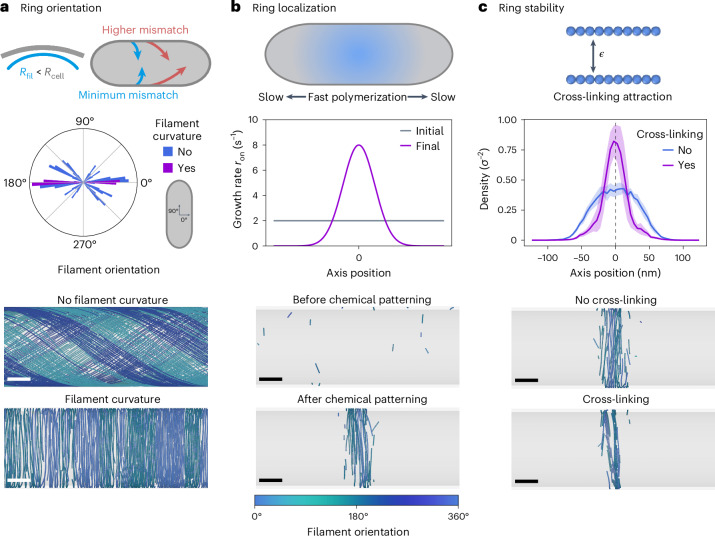
Formation of the bacterial division ring. **a**, Filament curvature and cell geometry drive the collective filament orientation along the cell circumference. Top, illustration of the curvature-sensing mechanism of FtsZ and FtsA filaments. Middle, measured distribution of filament orientations with and without filament curvature. Each bar plot corresponds to the average over *N* = 10 replicas in the nematic order region of the parameter space (*r*_on_ = 8 s^−1^, ${\tau }_{\det }=15\,{{{\rm{s}}}}$). Bottom, two representative snapshots of steady-state configurations of treadmilling filaments with and without filament curvature. **b**, Spatial modulation of FtsZ's growth and nucleation kinetics mediates its midcell localization. Top, illustration of the typical FtsZ kinetics modulation observed in vivo. Middle, example of a kinetics modulation combining an increase in the growth and nucleation rates around the midcell with a decrease at the poles. Bottom, two representative snapshots of system configurations before and after the modulation of the kinetics is switched on. **c**, Attractive interactions stabilize the ring and mediate tight packing. Top, illustration of the cross-linking implementation. Middle, late time (*t* > 8 minutes after the switch) density profiles along the cell axis with and without cross-linking interactions. The solid lines represent the mean over *N* = 10 replicas and the shaded region, the standard deviation. Bottom, two representative snapshots of steady-state rings with and without cross-linking interactions. In **b** and **c** we use switch parameters ${r}_{{{{\rm{on}}}}}^{0}=2\,{{{{\rm{s}}}}}^{-1}$, ${r}_{{{{\rm{on}}}}}^{1}=8\,{{{{\rm{s}}}}}^{-1}$ and *w*_prof_ = 100 nm for ${\tau }_{\det }=15\,{{{\rm{s}}}}$, where *w*_prof_ defines the profile width (see Supplementary Information). In all snapshots, the filaments are coloured according to their orientation with respect to the cell circumference (see colour bar at the bottom). For all simulations, we set *f*_curv_ = 5k_B_*T*/*σ*, *l*_p_ = 10 μm and *D* = 100 nm^2^ s^−1^ in a box of size *L* = 200 *σ* = 1 μm. Scale bars, 50 nm.

To showcase the importance of the identified self-organization mechanism in living cells, we investigate the role of treadmilling for bacterial division ring formation. Our in vivo experiments recently showed that treadmilling dynamics of FtsZ is essential for Z-ring formation in division of *B. subtilis*^23^, but the details linking the two remain elusive. We now use our coarse-grained molecular model for treadmilling filaments to explore the underlying mechanism. Bacterial FtsZ has been observed to display mild natural curvature in live cells and in vitro, with diameters similar to or smaller than typical cell diameters^13,32,49,50^. In addition, its membrane anchor FtsA—which makes composite polymers with FtsZ—forms intrinsically curved filaments that therefore display a preference for curved surfaces^51^. This suggests that the interplay between cell geometry and curvature of the (composite) filament should favour filament alignment along the circumference of the cell, similar to what has been previously shown for MreB filaments^52–54^. We incorporate this curvature-sensing mechanism in the model, as shown in Fig. 3a, by adding a circumference aligning force *f*_curv_ to the head and tail monomers of each filament (Supplementary Information). We find that a small force of this kind (*f*_curv_ = 5k_B_*T*/*σ*, where k_B_ is the Boltzmann constant and *T* is the temperature) is sufficient to determine the global orientation of the filaments along the cell circumference (Fig. 3a), highlighting the importance of the interplay between filament and cellular geometry in supramolecular organization.

Bacteria have evolved a number of different molecular tools that act in complementary ways to position FtsZ filaments to the right division site—the midcell—at the right time. Such systems include dynamic chemical patterning^55–60^ and condensate formation^61^ or nucleoid occlusion^62–65^ and generally have the effect of spatially modulating FtsZ polymerization, resulting in higher density of filaments at the midcell and lower density around the poles (schematic in Fig. 3b). This effect can be captured by incorporating higher polymerization rates at the midcell and lower rates around the poles. Our model can incorporate this FtsZ growth modulation as an instantaneous change from a uniform distribution along the cell body to a Gaussian profile centred at the midcell for growth and nucleation rates, with a constant detachment time (Fig. 3b and Supplementary Information). We find that the resulting combination of midcell enrichment and cell pole depletion of FtsZ proteins reliably localizes the filaments to the midcell region (Fig. 3b).

Finally, we consider one more model ingredient. It has been shown that FtsZ self-interaction, as well as cross-linking by FtsZ-binding proteins such as ZapA, can promote Z-ring condensation and that cross-linker deletion can lead to axial spreading of the rings^25,36,66,67^. In line with this, we find that including explicit attractive interactions between filaments has an important stabilizing effect on the ring structure, further promoting filament alignment and tight packing and allowing for a sustained monomer accumulation in the ring over time (Fig. 3c and Supplementary Fig. 18). Taken together, these results show that treadmilling filaments in our model can spontaneously form stable ring-like structures when a combination of filament curvature, attractive interfilament interactions and spatio-temporal modulation of the filament kinetics are considered.

### Comparison with in vivo data

**Fig. 4:**
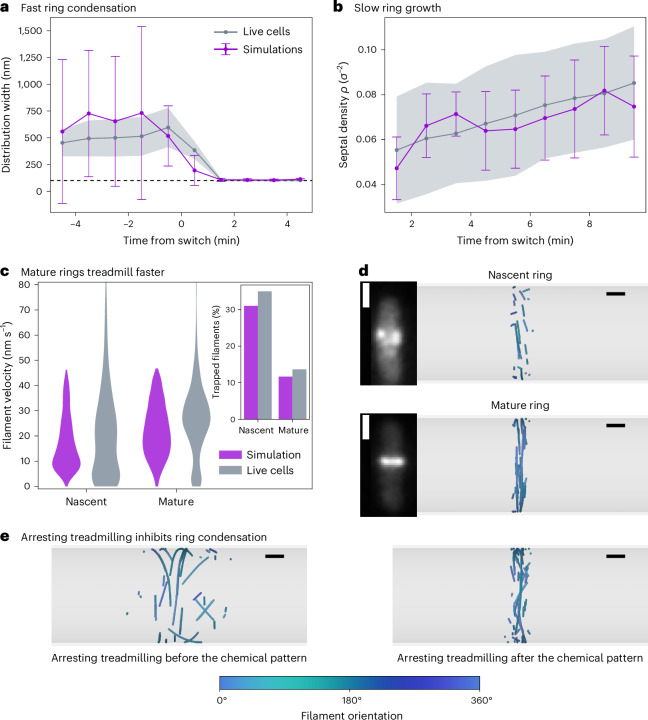
Z-ring formation in vivo. **a**, Z-ring condensation dynamics for live cells (grey) and simulations (purple). A rapid ring collapse around *t* = 0 minutes is observed. **b**, Ring density in time for live cells (grey) and simulations (purple). A positive average slope is observed, indicating the slow accumulation of proteins to the division site. In **a** and **b**, solid lines correspond to the average over samples (*N* = 10 replicas for simulations, *N* = 67 cells analysed from three independently prepared bacterial samples) and the shaded region or error bars to the standard deviation. Time *t* = 0 corresponds to the onset of the chemical pattern. **c**, Filament velocity distributions in nascent and mature rings for live cells (grey) and simulations (purple). Inset, fraction of trapped filaments (*v* < 10 nm s^−1^) for each distribution. In simulations we define *t* < 2 min for nascent and *t* > 8 min for mature rings. Statistics are done over *n* = 1,834 data points (filaments) for simulated nascent rings, *n* = 2,347 data points (filaments) for simulated mature rings, *n* = 1,066 data points for nascent in vivo rings and *n* = 3,126 data points for mature in vivo rings (note that this is the number of filaments imaged but the number of cells is *N* = 67 biological replicates). **d**, Representative snapshots of two successive ring configurations observed in vivo and in simulations. Microscopy images show fluorescently tagged FtsZ in live *B. subtilis* cells. These experiments were repeated independently with similar results. Simulations were repeated for *N* = 10 replicas with similar results. **e**, Representative snapshots of systems at *t* = 10 min after arresting treadmilling. Treadmilling is arrested 5 min before (left) and 1 min after (right) the onset of the chemical pattern that modulates the growth kinetics. The simulation data in **a**, **b** and **c** correspond to a switch with parameters ${r}_{{{{\rm{on}}}}}^{0}=2\,{{{{\rm{s}}}}}^{-1}$, ${r}_{{{{\rm{on}}}}}^{1}=9\,{{{{\rm{s}}}}}^{-1}$ and ${r}_{{{{\rm{nuc}}}}}^{1}=2\,{{{{\rm{s}}}}}^{-1}$ for ${\tau }_{\det }=15\,{{{\rm{s}}}}$ and *w*_prof_ = 100 nm. Note that monomers in simulations are rendered with size 20 nm instead of the actual 5 nm for visualization purposes and are coloured according to their orientation with respect to the cell circumference (see the colour bar at the bottom). Scale bars, 1 μm (**d**, insets), 200 nm (**d**,**e**).

We are now in a good position to compare our model for Z-ring formation with live-cell data. Our experiments have previously directly identified a very particular dynamics of Z-ring formation in living *B. subtilis* cells: localization of the ring to the midcell, often referred to as ring condensation, occurs very rapidly, within one minute (Fig. 4a), while ring maturation (growth and thickening) occurs over several minutes (Fig. 4b). If treadmilling is impaired early on, the Z-ring cannot form and remains diffuse, while impairing treadmilling during maturation disrupts proper division^23^. Our experiments also found that the average treadmilling velocity of filaments in mature Z-rings is substantially faster than in nascent ones (Fig. 3c).

To explore whether our model can capture the dynamics of Z-ring formation in vivo and explain the mechanism behind it, we simulate systems on the cell scale (*L* = 3 μm giving *R* ≈ 1 μm). We limit the total amount of monomers to the estimated amount of FtsZ in *B. subtilis* Z-rings ${N}_{\max }=2,000$ (Supplementary Information), resulting in a relatively low monomer surface density. Strikingly, as shown in Fig. 4a and Supplementary Video 8, our model also displays rapid ring condensation, which occurs only a few seconds after the switch of the modulation profile. This behaviour is caused by the rapid dying out of the filaments in the growth-inhibited areas of the cell, as their depolymerization rate is faster than the polymerization rate. The whole process occurs on the scale of the single monomer’s lifetime, as controlled by ${\tau }_{\det }$. Hence, treadmilling allows the system to rapidly respond to external chemical cues.

We further find, in agreement with live-cell experiments, that after rapid ring condensation at the site of division, treadmilling filaments in the model accumulate rather slowly, over a time-span of several minutes. The simulations reveal a steadily increasing surface density of monomers in the midcell region that matches well the experimental measurements (Fig. 4b). This feature is the inherent result of treadmilling-driven alignment: the ring structure transitions from an initially disordered state to an ordered state populated by several bundles of filaments tightly bound together (Fig. 4d and Supplementary Video 8) through the dissolution and replacement of misaligned filaments in a process that is analogous to what we observed in vitro for *E. coli* FtsZ. Mature rings are thus composed of small patches of FtsZ organized in bands that are a few filaments wide and travel together, as previously observed in live-cell imaging^19,68,69^ (Fig. 4d and Supplementary Video 8). These bands are tightly bound, displaying high local density of monomers, consistent with cryotomography experiments estimating an interfilament distance of approximately 6 nm in FtsZ rings^70^.

Ring maturation is driven by the replacement of misaligned filaments, which is at heart a trial-and-error process, rendering this process slow. The decrease in the fraction of low-velocity misaligned filaments in the mature ring also naturally leads to faster treadmilling velocities without individual filaments speeding up, which matches live-cell measurements (Fig. 4c). In other words, the surviving filaments all run parallel to each other with velocities close to maximal values. Finally, we found that arresting treadmilling in our model has similar effects to those observed in vivo. If treadmilling is arrested early on, before the onset of the chemical patterning, the filament population remains diffuse and never condenses to the midcell (Fig. 4e and Supplementary Video 9). If treadmilling is arrested after the switch of the chemical patterning, the ring forms at the midcell, but displays aberrant, ‘frozen’ configurations (Fig. 4e, Supplementary Fig. 21 and Supplementary Video 10). The crucial role of treadmilling in the positioning of the FtsZ ring that our model reveals has also been previously discussed in the context of *E. coli*: it has been reported that mutants that are insensitive to classical positioning systems like Min (FtsZ2, FtsZ9 and FtsZ100) all have very low or undetectable GTPase activity. This implies that the lack of treadmilling underlies their inability to correctly position the ring^28^.

Altogether, our treadmilling model naturally reproduces the key dynamical aspects of Z-ring formation observed in live cells. FtsZ filaments are able to localize to the midcell on timescales similar to the monomer lifetimes, driven by the underlying spatial modulation of the polymerization rates, while the tightly packed division rings grow on substantially longer timescales by virtue of misaligned filaments dying and growing again until only the aligned ones remain. This mechanism is at play irrespective of the filament density: simulations of treadmilling filaments on a surface constrained to in-vivo-like low protein concentrations show that ordering still occurs and is driven by the same mechanism of death by misalignment (Supplementary Fig. 14). More generally, our model explains the importance of treadmilling—dynamic growth and shrinkage of filaments—in localizing and timing bacterial division.

## Discussion

Our treadmilling model reveals a surprisingly rich collective behaviour arising from the dissolution and replacement of misaligned filaments, characteristic of mortal polymers. These findings put forward treadmilling filaments as a paradigm for a dynamic self-ordering system that is able to quickly remodel itself in response to external cues. This suggests that both naturally occurring and artificial treadmilling polymers could be a useful tool in areas such as synthetic cell development or programmable active matter, capable of healing via local filament dissolution. For example, microfluidic devices and chemical or photo patterning could be used experimentally to generate and control dynamic treadmilling filament systems.

From a biological perspective, our model provides structural and dynamic insights into functional FtsZ assemblies. The model can further be used to investigate how the dynamics of the Z-ring affects recruitment and activation of bacterial wall synthesis machinery, resulting in the growth of the cell wall at midcell, which ultimately divides the cell into two^15,16,71–73^.

From a physics point of view, we find that treadmilling filaments belong to a distinct class of active matter, which consumes energy not for motility but for turnover and thus order via death of locally misaligned filaments. This mechanism is expected to have further implications for filament collective behaviour, such as the response to obstacles, perturbations and propagation of information. For instance, treadmilling filaments can be expected to evacuate the region around obstacles due to their mortality, instead of being trapped. Due to filament dissolution, such systems are also expected to locally heal upon defect introduction or external perturbation and are not expected to transduct the perturbation deep into the assembly. Overall, by revealing a distinct ordering mechanism in chemically active matter, our work highlights the importance of exploring the collective and material properties of explicitly mortal filaments.

### Reporting summary

Further information on research design is available in the Nature Portfolio Reporting Summary linked to this article.

## Online content

Any methods, additional references, Nature Portfolio reporting summaries, source data, extended data, supplementary information, acknowledgements, peer review information; details of author contributions and competing interests; and statements of data and code availability are available at 10.1038/s41567-024-02597-8.

## Supplementary information

Supplementary Information Supplementary Figs. 1–21, Methods and extended results and discussion and Videos 1–10.

Reporting Summary

Supplementary Video 1

Supplementary Video 2

Supplementary Video 3

Supplementary Video 4

Supplementary Video 5

Supplementary Video 6

Supplementary Video 7

Supplementary Video 8

Supplementary Video 9

Supplementary Video 10

## Data Availability

The simulation data presented in this work are available from the University College London public data repository at 10.5522/04/24754527 (ref. ^74^). Live-cell imaging of FtsZ rings data presented in this work are from ref. ^23^. High-speed atomic force microscopy data presented in this work are from ref. ^9^. Total internal reflection fluorescence microscopy data presented in this work are from refs. ^37,75,76^.

## Code availability

Appropriate documentation and example files to replicate the simulation results presented in this work are available from the University College London public data repository at 10.5522/04/24754527 (ref. ^74^). A maintained version of the code is available on GitHub^77^. Simulation data analysis was performed using custom Python code, which makes use of several public libraries such as NumPy, pandas or Ovito, also available at ref. ^74^. ImageJ (current version v.1.54) and Python (current version Jupyter Notebook v.6.5.4) were used for the in vitro data analysis. Live-cell imaging videos were analysed using Fiji (v.1.53 and v.1.54) with open-source plugins PureDenoise, StackReg, MicrobeJ (v.5.13I), ilastik (v.1.3.3post2) and custom code available via GitHub at https://github.com/HoldenLab/Ring_Analysis_IJ). Further in vivo data analysis was done using Matlab with custom code available via GitHub at https://github.com/HoldenLab/ring-fitting2; https://github.com/HoldenLab/violinplusDABEST-Matlab and https://github.com/HoldenLab/ring-simulator.
