# Supplementary material for: Self-organization of mortal filaments and its role in bacterial division ring formation
Source: Nat Phys. 2024 Aug 12;20(10):1670–8. doi: 10.1038/s41567-024-02597-8 (PMC11473364; doi:10.1038/s41567-024-02597-8)
Supplement: Supplementary file 2 — Reporting Summary [file 41567_2024_2597_MOESM2_ESM.pdf]

Corresponding author(s): Anđela Šarić
Last updated by author(s): Jun 5, 2024

Reporting Summary

Nature Portfolio wishes to improve the reproducibility of the work that we publish. This form provides structure for consistency and transparency in reporting. For further information on Nature Portfolio policies, see our Editorial Policies and the Editorial Policy Checklist.

Statistics

For all statistical analyses, confirm that the following items are present in the figure legend, table legend, main text, or Methods section.

n/a	Confirmed	
☐	☒	The exact sample size (n) for each experimental group/condition, given as a discrete number and unit of measurement
☐	☒	A statement on whether measurements were taken from distinct samples or whether the same sample was measured repeatedly
☒	☐	The statistical test(s) used AND whether they are one- or two-sided<br>*Only common tests should be described solely by name; describe more complex techniques in the Methods section.*
☒	☐	A description of all covariates tested
☒	☐	A description of any assumptions or corrections, such as tests of normality and adjustment for multiple comparisons
☐	☒	A full description of the statistical parameters including central tendency (e.g. means) or other basic estimates (e.g. regression coefficient) AND variation (e.g. standard deviation) or associated estimates of uncertainty (e.g. confidence intervals)
☒	☐	For null hypothesis testing, the test statistic (e.g. F, t, r) with confidence intervals, effect sizes, degrees of freedom and P value noted<br>*Give P values as exact values whenever suitable.*
☒	☐	For Bayesian analysis, information on the choice of priors and Markov chain Monte Carlo settings
☒	☐	For hierarchical and complex designs, identification of the appropriate level for tests and full reporting of outcomes
☒	☐	Estimates of effect sizes (e.g. Cohen's d, Pearson's r), indicating how they were calculated

Our web collection on statistics for biologists contains articles on many of the points above.

Software and code

Policy information about availability of computer code

Data collection

All the computational data presented in this work was obtained by running Molecular Dynamics simulations on a custom modified version of the LAMMPS software package (September 2021 release). A maintained version of this custom modifications together with some Python scripts to generate the required input files to run the simulations are available for download at the following GitHub repository: https://github.com/Saric-Group/treadmilling -- A copy of the current version of this code is also available at the following UCL public data repository: https://doi.org/10.5522/04/24754527 -- Note that this repository will only be activated upon publication of the work. Both of these repositories also include all necessary instructions to replicate the data presented in this work

Data analysis

Simulation data analysis was performed using custom Python code which makes use of several public libraries such as numpy, pandas or ovito. All necessary code to replicate the analysis performed for this work is available for download at the following UCL public data repository: https://doi.org/10.5522/04/24754527

ImageJ (current version 1.54) and Python (current version Jupyter Notebook 6.5.4) was used for the in vitro data analysis.

Live cell imaging videos were analysed using Fiji (v1.53 and v1.54) with open-source plugins PureDenoise, StackReg, MicrobeJ (v.5.13I), and ilastik (v.1.3.3post2); and custom code available on the Holden lab Github page: https://github.com/HoldenLab/Ring_Analysis_IJ. Further in vivo data analysis was done using Matlab with custom code available on the Holden lab GitHub page:
- https://github.com/HoldenLab/ring-fitting2
- https://github.com/HoldenLab/violinplusDABEST-Matlab
- https://github.com/HoldenLab/ring-simulator

For manuscripts utilizing custom algorithms or software that are central to the research but not yet described in published literature, software must be made available to editors and reviewers. We strongly encourage code deposition in a community repository (e.g. GitHub). See the Nature Portfolio guidelines for submitting code & software for further information.

Data

Policy information about availability of data

All manuscripts must include a data availability statement. This statement should provide the following information, where applicable:

- Accession codes, unique identifiers, or web links for publicly available datasets
- A description of any restrictions on data availability
- For clinical datasets or third party data, please ensure that the statement adheres to our policy

All the necessary codes to replicate and analyse the simulation data presented in this work is available for download at the following UCL public data repository: https://doi.org/10.5522/04/24754527 -- Note that this repository will only be activated upon publication of the work, if you need to access it before we can provide a private link via email.

Live cell imaging of FtsZ rings data presented in this work is from Whitley, K. et al., Nat. Comms. 2021 (doi: 10.1038/s41467-021-22526-0).

High-Speed Atomic Force Microscopy data presented in this work is from Dunajova, Z. et al., Nat. Phys. 2023 (doi: 10.1038/s41567-023-02218-w).

TIRF microscopy data presented in this work is from:
1. Baranova, N. and Loose, M., Methods in Cell Biology, Academic Press 2017 (doi: 10.1016/bs.mcb.2016.03.036)
2. Caldas, P. et al., Methods in Cell Biology, Academic Press 2020 (doi: 10.1016/bs.mcb.2020.01.006)
3. Baranova, N. et al., Nat. Microbio. 2020 (doi: 10.1038/s41564-019-0657-5)

Human research participants

Policy information about studies involving human research participants and Sex and Gender in Research.

Reporting on sex and gender	No human or animal participants were involved in this research
Population characteristics	No human or animal participants were involved in this research
Recruitment	No human or animal participants were involved in this research
Ethics oversight	No human or animal participants were involved in this research

Note that full information on the approval of the study protocol must also be provided in the manuscript.

Field-specific reporting

Please select the one below that is the best fit for your research. If you are not sure, read the appropriate sections before making your selection.

☒ Life sciences ☐ Behavioural & social sciences ☐ Ecological, evolutionary & environmental sciences

For a reference copy of the document with all sections, see nature.com/documents/nr-reporting-summary-flat.pdf

Life sciences study design

All studies must disclose on these points even when the disclosure is negative.

Sample size	Simulation data: Replica numbers were chosen to guarantee sufficient sampling of stochastic processes and were limited by computational cost.<br><br>In vitro data: Sample sizes were not chosen according to a statistical test, but were limited by practicality and throughput<br><br>In vivo data: No a priori sample size calculations were performed. No specific sample size was chosen as the single cell / single molecule nature of the measurements means moderate to large sample size, sufficient for robust statistical analysis, is usually straightforward to achieve.<br><br>In general, numbers of data points, simulation replicas and experimental counts are always specified in figure captions appropriately.
Data exclusions	Simulation data: No data was excluded from analysis.<br><br>In vitro data: Images were only excluded when supported lipid bilayers were defective.<br><br>In vivo data: In total 42 Z-rings (6%) were manually excluded due to failure of the joint septal localisation and background subtraction algorithm.
Replication	Simulation data: simulations were run several times (see sample size numbers in manuscript) for each condition described, each with a

Replication	different random number generator seed to guarantee proper sampling of the stochastic processes simulated.<br><br>In vitro data: The results were verified and reproduced with different batches of purified FtsZ and FtsA proteins. 5 different experiments were analysed in this work.<br><br>In vivo data: Z-ring imaging experiments were repeated 3 times using independently-prepared samples.
Randomization	Allocating experimental groups was not relevant for this study as only the Z-ring data involves living organisms and in this case all bacterial cells of a particular strain are genetic clones.
Blinding	Blinding was neither possible nor necessary for this study as only the Z-ring data involves living organisms and in this case 1) all bacterial cells of a particular strain are genetic clones and 2) analyses were not sufficiently subjective to require researcher blinding.

Reporting for specific materials, systems and methods

We require information from authors about some types of materials, experimental systems and methods used in many studies. Here, indicate whether each material, system or method listed is relevant to your study. If you are not sure if a list item applies to your research, read the appropriate section before selecting a response.

Materials & experimental systems

n/a	Involved in the study
☒	☐ Antibodies
☒	☐ Eukaryotic cell lines
☒	☐ Palaeontology and archaeology
☒	☐ Animals and other organisms
☒	☐ Clinical data
☒	☐ Dual use research of concern

Methods

n/a	Involved in the study
☒	☐ ChIP-seq
☒	☐ Flow cytometry
☒	☐ MRI-based neuroimaging
